# Remarkable Case of Abdominal Tumor

**Published:** 1844-06

**Authors:** 


*Remarkable Case of Abdominal Tumor.*—Mr. Dendy, after some comments on the acknowledged difficulty in the diagnosis of abdominal tumor, read a case, of which we give the following extract:

A gentleman, 31 years of age, previously in fair health, yet having suffered for a week under severe influenza, and for which he had taken large doses of nitrate of potash, was suddenly attacked, on the 10th of October, by abdominal tumefaction of the right side with but very slight concomitant uneasiness. The tumor increased so rapidly that within twenty hours from its perceptible commencement it reached from the margin of the ribs to Poupart's ligament, extending across the umbilicus, projecting into the loins, and resting on the crista of the left ilium. It was then insensible to pain on pressure, yet producing dyspnœa from its encroachment on the diaphragm, &c.

After a few days, during which there was complete constipation, neuralgic pain supervened, first in the lumbar plexus and afterwards in the sacral, and the nerves proceeding from them. This neuralgic pain became, in the course of the disease, occasionally so agonizing as to produce extreme agitation, and subsequently stupor. The pulse and urinary secretion were nearly natural.

Enemata, repeated purgatives, and anodynes were administered. The bowels were not relieved for nearly ten days, and then for nearly a week longer the evacuations, even after enemata, though the elastic tube was introduced as far, at least, as the upper angle of the sigmoid flexure were merely morbid grumous secretions of the intestine.

About the 30th of October the evacuations became more feculent, but mixed with numerous shreds of coagulated mucus, resembling grape-skins. Emaciation was now visible, and this progressively increased, notwithstanding a very fair proportion of nutrition was administered and retained, and various medicines were exhibited. Early in November colocynth and compound decoction of aloes succeeded, after a day or two, in producing more feculent motions. Still the tumor increased until the abdomen measured two feet six inches in circumference.

On November 20th pus began to flow with the urine in large quantity, and was attended by very perceptible diminution of the tumor; still emaciation continued until the end of November. From this period the patient gained flesh, and on the 6th of December, the urine having become almost natural, he was pronounced convalescent.

The interest excited regarding this case was increased by the circumstance of this being the second severe attack, marked by precisely the same symptoms; the first appearing four years since. He had, however, suffered under three other very transient attacks in early life, at almost regular periods, which speedily yielded to purgatives.

Many acute pathologists visited this gentleman in the course of the malady—their diagnosis differing considerably—the principal opinions referring to colic, impaction, congested spleen, melanotic, or even malignant growth, serous cysts attached to the peritoneum, or developed in submucous tissue, and suppuration of the kidney in its capsule.

The author analysed these and other notions suggested during attendance, referring to the absence of records of analogous cases in Morgagni, &c., and alluding to other cases recorded by Dr. James Johnson many years since, leaving the essential or primary nature of the disorder, whether consisting in pyelitis, with masked symptoms, or in cystic suppuration bursting into the collapsed capsule of the kidney, as well as the etiology of the case, especially with reference to the nitrate of potash, as an open question for discussion.

Dr. James Johnson had lived long enough in the world not to be ashamed to acknowledge an error. The result of the case nuder discussion proved that he had been wrong in suspecting it to be dependent upon disease of the spleen. He was now of opinion that the tumor had resulted from distention of the pelvis of the kidney; and he handed to the secretary an abstract of a case of renal suppurative dropsy which had come under his care sometime since. The subject of the case was a woman, eight months advanced in pregnancy. She complained of violent pain in the right side of the abdomen, extending from the umbilicus round to the lumbar vertebræ, and from the floating ribs to the groin. She had fever, with scanty, highly colored urine. The abdomen appeared unusually large, and there was a distinct *sulcus* running from the scrobiculus cordis to the pubis, on each side of which the abdominal tumor rounded out as though there were two impregnated wombs. She reported that of late her urine was sometimes scanty, sometimes copious and white like milk. On the 10th of May she fell into labour, and next day was delivered of a living child. The right side of the abdomen remained as large as before delivery, presenting a considerable tumor, seven or eight inches in diameter, perfectly smooth on its surface and giving the feel of fluctuation. Fever, colliquative diarrhœa, and constant micturition followed. On the 20th of May the nurse was surprised to find the bed, and even the floor of the room covered with a milky fluid, which had escaped in the night, without the knoweledge of the patient, who had slept soundly. The tumor was gone. Fever, however, and diarrhœa continued, and she sunk in a few days. After death a large cyst was found, which, on being opened, discharged about three pints of milky fluid. The whole internal surface of the bag was very vascular, and thickly studded with a kind of papillary bodies, varying in size from that of a pin's-head to a pea. The ureter, of enormous size in some places, was traced from the cyst into the bladder, in which last viscus there was about a pint of this milky or muco-purulent fluid. No part of the substance of the kidney, but only this great capsule could be found. The cyst, on calculation, would probably contain about five or six quarts of fluid. She had had a similar attack in a former pregnancy, which subsided after delivery, on the supervention of a large quantity of milky fluid. The post-mortem examination of this case was conducted very quickly, and under unfavorable circumstances. No stricture of the ureter could be detected, but he had little doubt that it really did, or had, existed, as this canal was in some parts as large as the aorta. Pregnancy had produced this stricture by pressure. He thought that there was a strong analogy between this case and the one related by Dr. Dendy.

Mr. Pilcher observed that when he saw Mr. Dendy's patient in the attack under which he labored four years since, he had no doubt at the time that the tumor was intestinal; others agreed with him in this opinion, and hence he was induced to recommend the introduction of a long tube into the intestines. The second attack was of a similar character, and the same plan was recommended. He was probably led to form this opinion from having dissected a case attended by similar symptoms, and which was supposed to be disease of the kidney, chiefly, from the circumstance of the urine containing pus on more than one occasion. On examination, all the urinary organs were found healthy. No marks whatever of a tumor could be detected; she died of diarrhœa. In this case, he had no doubt that the tumor had consisted of a collection, probably of fluid, in the colon, and that the discharge of pus from the bladder was merely a coincidence.

In the second attack, in Mr. Dendy's case, it was considered that the tumor was situated, probably, in the ileum, and that as it became diminished by the discharge per anum, it became refilled by the secretion of fresh fluid. The discharge of large quantities of gelatinous matter somewhat favored this conjecture, as this matter was supposed to be secreted by the mucous membrane of the intestines. Other circumstances in favor of this opinion were the sensation the patient experienced on the introduction of the pipe, of its having passed *behind* the tumor, the constipation previous to the attack, the subsequent discharges, and the severe neuralgia, which was evidently the result of the pressure exerted by the tumor. Dr. Bright had felt no doubt but that the first attack was a well-marked instance of suppuration of the kidney. It was certain that in proportion as the pus was secreted from the bladder, the tumor diminished in size; but it must be recollected that there was diarrhœa, also, and it was difficult to say upon which discharge the diminution of the tumor depended. Dr. Bright had been surprised at the occurrence of the second attack, as he supposed that the kidney had suppurated entirely away, but considered that the cyst which would have remained had become inflamed and suppurated. The opinion of the speaker was, that the cyst of the kidney had remained in a collapsed state after the subsidence of the first attack; but on the occurrence of a general disordered state of the health, or the determination of a large quantity of blood to the cyst, the disease had returned. The periodic nature of the attacks was a point of much interest; the first occurred at seven, the second at fourteen, the third at twenty-one, and the fourth at twenty-eight years of age; there had been an interval of only four years between this and the last attack. Cases similar to that of Mr. Dendy were of uncommon occurrence, except as the result of stricture, or some mechanical obstruction to the flow of urine, from which originated a collection of fluid in the pelvis of the kidney. It was possible that stricture might exist in Mr. Dendy's case, but he did not think that it did.

Dr. T. Thomson considered the disease, in Mr. Dendy's case, to have consisted of suppuration in one or more of the calyces, or of the entire pelvis, of the kidney. The cause was obscure. He related a case of a woman subject to diarrhœa, and who had a large tumor on the left side of the abdomen. Pus was found in the urine, and occasionally the triple-phosphate deposit. With a view ot dissolving this, small doses of hydrochloric acid were administered, but were obliged to be discontinued in consequence of its severe action on the bowels. She was subject to severe pains, which were relieved by the administration of morphia. She lived two or three years, and after death the left kidney was found very much enlarged, and filled with pus. There was a triple-phosphate calculus impacted in the ureter of that side. Cases similar to Mr. Dendy's were usually the result of obstruction in the ureter; there was no evidence, however, of such an obstruction in this case. The long interval between the attacks favored the opinion. In Mr. Dendy's case there was no deposit of triple-phosphate in the urine.

Dr. L. Stewart related a case, in which the tumor, after death, was found to consist of an enlarged and thickened bladder.

Mr. Dendy, in answer to questions, stated that the patient whose case he had detailed, had, previous to the first attack of disease, taken a drachm of nitrate of potash twice a day; and two drachms three times a day previous to the last attack. This he did in opposition to his medical adviser.

Dr. Johnson considered this quantity sufficient to do mischief to the kidneys, and wondered it had not also seriously affected other organs. He had seen half-drachm doses very injurious to the stomach.

Dr. Willshire suggested that the nitrate of potash had acted as a powerful stimulant in this case, producing inflammation, and consequent suppuration of the kidney.

Mr. Headland observed that the value of the case under discussion much depended on the advantage it afforded of diagnosticating any similar case, should such be presented to our notice. He thought its whole history proved that the disease could not have been situated in the colon or the spleen, neither did he think it was at all proved that in the last case the kidney had suppurated. We had certainly no evidence of the presence of nephritis, in the usual sense of that term, the more prominent symptoms of that disease being absent. He was inclined, therefore, to think that there had been suppuration of the kidney in one of the former attacks, and that the cyst left after this disease had, in the last attack, become filled with fluid, not exactly pus, but an elimination from the blood. Did this arise from the administration of the nitre? We knew that in large doses this medicine was injurious, and did not act as a diuretic unless largely diluted with water. He therefore thought that in the present case the first effect of the medicine had been on the bowels, producing the peculiar evacuations which had been mentioned. It then appeared to have excited such an action on the cyst as to have caused an elimination from the blood to be secreted in it

There were no signs or symptoms of the formation of pus. If inflammation had existed to such an extent as it must have done to produce suppuration, the patient most likely would have succumbed.—*London Lancet*, for January.

